# Clinical and Molecular Spectrum of *PPP2R1A*-Related Neurodevelopmental Disorders: A Systematic Review

**DOI:** 10.3390/genes16121508

**Published:** 2025-12-16

**Authors:** Jaewoong Lee, Ari Ahn, Jaeeun Yoo, Seungok Lee

**Affiliations:** Department of Laboratory Medicine, Incheon St. Mary’s Hospital, College of Medicine, The Catholic University of Korea, Incheon 21431, Republic of Korea; 0124ari@catholic.ac.kr (A.A.); focused@catholic.ac.kr (J.Y.); lsok@catholic.ac.kr (S.L.)

**Keywords:** *PPP2R1A*, Houge-Janssens syndrome 2, neurodevelopmental disorder, intellectual disability, epilepsy, corpus callosum agenesis, genotype-phenotype correlation

## Abstract

**Background/Objectives**: *PPP2R1A* encodes the scaffold subunit Aα of protein phosphatase 2A (PP2A). Pathogenic variants cause Houge-Janssens syndrome 2, a rare neurodevelopmental disorder characterized by developmental delay, intellectual disability, epilepsy, and brain malformations. We systematically reviewed published cases to define the clinical spectrum, characterize the mutational landscape, and explore genotype–phenotype correlations. **Methods**: We conducted systematic searches of PubMed, Embase, and Web of Science from inception to March 2025, supplemented by GeneReviews and OMIM references. Studies reporting *PPP2R1A* variants with clinical data were included. Data extraction followed PRISMA guidelines, encompassing study characteristics, genetic findings, and phenotypic features. **Results**: We identified 16 studies representing 60 patients with *PPP2R1A*-related disorders. Twenty-six distinct pathogenic variants were identified; these were predominantly de novo heterozygous missense changes clustering within HEAT repeats 5–7. Recurrent hotspots included p.Arg182Trp (*n* = 12) and p.Arg183Gln (*n* = 5). Developmental delay and intellectual disability were universally present in all patients for whom data were available (100%, 58/58). Epilepsy occurred in 50.9% (29/57), and structural brain abnormalities in 83.1% (49/59), with corpus callosum abnormalities (40.7%, 24/59) and ventriculomegaly (32.2%, 19/59) being most frequent. Microcephaly was reported in 17.2% (10/58) and macrocephaly in 25.9% (15/58), while dysmorphic features were present in 53.4% (31/58). The phenotypic spectrum ranged from severe neonatal presentations with high mortality to milder neurodevelopmental courses, with prenatal manifestations including ventriculomegaly, corpus callosum abnormalities, and rare cardiac defects. Clear genotype–phenotype correlations emerged, with HEAT5 variants (p.Arg182Trp, p.Arg183Gln) associated with severe phenotypes and increased mortality, while p.Arg258His variants demonstrated comparatively milder courses. **Conclusions**: *PPP2R1A*-related disorders encompass a broad clinical spectrum ranging from lethal neonatal disease to survivable forms with variable neurodevelopmental outcomes. Prenatal features including ventriculomegaly and corpus callosum abnormalities enable early genetic diagnosis, informing reproductive counseling. Recognition of recurrent hotspot variants and their phenotype associations facilitates diagnosis, prognosis, and genetic counseling. These findings provide evidence-based guidance for clinical management and highlight the importance of variant-specific prognostication in this emerging neurodevelopmental disorder.

## 1. Introduction

Neurodevelopmental disorders (NDDs) are a heterogeneous group of conditions characterized by global developmental delay, intellectual disability, epilepsy, and structural brain abnormalities [1,2]. With the widespread application of next-generation sequencing technologies, an increasing number of novel genes have been implicated in NDDs [3,4]. Despite these advances, many causative genes remain incompletely characterized, and the phenotypic spectrum of several recently described disorders continues to expand [1].

The *PPP2R1A* gene, which encodes the scaffold subunit Aα of the protein phosphatase 2A (PP2A) holoenzyme, has emerged as an important contributor to severe neurodevelopmental phenotypes [1,5]. PP2A is a highly conserved serine/threonine phosphatase complex involved in numerous cellular processes, including signal transduction, cell cycle regulation, and brain development [6,7]. PPP2R1A provides the structural framework for the assembly of the catalytic (C) and regulatory (B) subunits, with pathogenic variants typically disrupting holoenzyme stability or subunit binding, leading to distinct clinical manifestations [1,5].

The PP2A holoenzyme is a heterotrimeric complex consisting of three subunits: a catalytic C subunit (PPP2CA or PPP2CB), a scaffolding A subunit (PPP2R1A or PPP2R1B), and a regulatory B subunit (multiple gene families including PPP2R5A-E, PPP2R2A-D, and others) [1,7,8]. The scaffolding A subunit, encoded by *PPP2R1A*, is composed of 15 tandem HEAT (Huntingtin, Elongation factor 3, PR65/A subunit, TOR) repeats that form a horseshoe-shaped structure providing the structural platform for C and B subunit binding [1,9]. Pathogenic variants in *PPP2R1A* typically cluster in HEAT repeats 5–7, a region critical for regulatory B subunit binding, thereby disrupting holoenzyme stability and function [1,5].

Notably, pathogenic variants in multiple PP2A complex genes cause overlapping neurodevelopmental phenotypes collectively termed PP2A-related disorders or Houge-Janssens syndrome spectrum. While *PPP2R1A* variants (Houge-Janssens syndrome 2, OMIM #616362) are associated with intellectual disability, hypotonia, language impairment, epilepsy, microcephaly, and brain malformations, variants in PPP2R1B (encoding the alternative Aβ scaffold) and PPP2R5D (encoding the B56δ regulatory subunit, Houge-Janssens syndrome 1) produce remarkably similar clinical features including intellectual disability, macrocephaly, hypotonia, and autism [9]. Additionally, pathogenic variants in other PP2A regulatory B subunits, including PPP2R5C, PPP2R5E, and PPP2R2B, have been associated with neurodevelopmental phenotypes featuring macrocephaly, intellectual disability, seizures, hypotonia, and specific learning disorders [10,11,12], further emphasizing the pivotal role of PP2A complex integrity in brain development.

Pathogenic *PPP2R1A* variants were first described in 2015 by Houge and colleagues, who identified de novo missense variants in patients with severe intellectual disability and structural brain abnormalities [6]. Since then, the disorder has been designated Houge-Janssens syndrome 2 (HJS2, OMIM #616362), with additional case reports and cohorts expanding the mutational spectrum and clinical features.

Reported phenotypes include global developmental delay, variable degrees of intellectual disability, epilepsy, corpus callosum agenesis or hypoplasia, ventriculomegaly, cerebellar or brainstem hypoplasia, hypotonia, and congenital heart defects [1,5]. Recent studies have identified distinct clinical subgroups based on head circumference, with macrocephalic patients showing milder presentations and microcephalic patients having higher epilepsy rates and more severe intellectual disability [3,13].

Recurrent *PPP2R1A* missense changes cluster in HEAT repeats 5–7, with hotspot variants including p.Arg182Trp, p.Arg183Gln, and p.Arg258His [6,14]. These variants typically disrupt holoenzyme stability or subunit binding, with specific molecular consequences correlating with clinical severity [1,15]. Functional studies demonstrate that variants affecting B-subunit binding correlate with more severe phenotypes.

Recent studies have highlighted prenatal presentations, including corpus callosum dysgenesis, ventriculomegaly, and hydrocephalus detected by fetal imaging. Prenatal exome sequencing has successfully identified *PPP2R1A* variants in affected fetuses, expanding the clinical recognition of the disorder to the prenatal period [16,17,18].

Despite these advances, the literature on *PPP2R1A*-related disorders remains fragmented, consisting primarily of isolated case reports and small series that limit comprehensive understanding of the clinical spectrum [14,19]. While previous narrative reviews have provided valuable insights [20], they have not systematically integrated the growing body of evidence or comprehensively examined genotype–phenotype correlations. Therefore, a systematic review is needed to synthesize all available evidence, define the full clinical spectrum, and establish robust genotype–phenotype relationships to guide clinical practice and genetic counseling.

Therefore, we conducted a systematic literature review of *PPP2R1A*-related neurodevelopmental disorders following the PRISMA guidelines to comprehensively define the clinical spectrum, characterize the mutational landscape, and explore genotype–phenotype correlations [21]. This review aims to provide evidence-based insights for clinical diagnosis, genetic counseling, and future research directions.

## 2. Materials and Methods

### 2.1. Search Strategy

This systematic review was conducted according to the guidelines of the Preferred Reporting Items for Systematic Reviews and Meta-Analyses (PRISMA) 2020 statement. The protocol for this review was not registered in a public database. We conducted systematic searches of PubMed, Embase, and Web of Science from database inception to March 2025, using a combination of controlled vocabulary terms and free-text keywords related to *PPP2R1A* and neurodevelopmental disorders. Complete search strategies were developed a priori and are provided in full in the Supplementary Material S1 Supplementary Methods. Representative search strings included:

PubMed: (“*PPP2R1A*” OR “protein phosphatase 2 regulatory subunit A alpha”) AND (“neurodevelopmental disorder” OR “intellectual disability” OR “epilepsy” OR “brain malformations”).

Embase: (‘ppp2r1a’/exp OR ‘ppp2r1a’:ab,ti) AND (‘neurodevelopmental disorder’/exp OR ‘intellectual disability’/exp OR ‘epilepsy’/exp OR ‘brain malformation’/exp).

Web of Science: TS = (“*PPP2R1A*” OR “protein phosphatase 2 regulatory subunit A alpha”) AND TS = (“neurodevelopmental disorder” OR “intellectual disability” OR “epilepsy” OR “brain malformation”).

We also screened the reference lists of GeneReviews^®^ and OMIM #616362 for additional primary case reports. The complete search strings for each database are provided in the Supplementary Material S1.

ClinVar was not included as a primary search database, as it aggregates variant-level data without the patient-level phenotypic details and detailed clinical descriptions required for systematic review of genotype–phenotype correlations.

### 2.2. Eligibility Criteria

Inclusion criteria: Human studies (case reports, case series, or cohort studies) reporting patients with pathogenic or likely pathogenic *PPP2R1A* variants according to ACMG/AMP guidelines, with detailed clinical phenotype descriptions and genetic variant information.

Exclusion criteria: Reviews, editorials, conference abstracts, animal or in vitro studies, studies focusing primarily on oncology without neurodevelopmental phenotypes, and studies reporting variants of uncertain significance (VUS) without supporting functional evidence.

### 2.3. Study Selection and Data Handling

Two reviewers (J.Y. and A.A.) independently screened titles and abstracts using predetermined eligibility criteria, followed by full-text evaluation of potentially relevant studies. Disagreements were resolved through discussion or consultation with a third reviewer (S.L.). Records were exported in RIS and CSV formats, deduplicated using PMID, DOI, and title matching, and pre-filtered using a standardized Python (v.3.11.2) pipeline with inclusion keywords (“developmental delay,” “intellectual disability,” “epilepsy,” “brain malformation”) and exclusion terms (oncology-related keywords). All automated filtering decisions were manually verified.

### 2.4. PRISMA Summary

**Figure 1:**
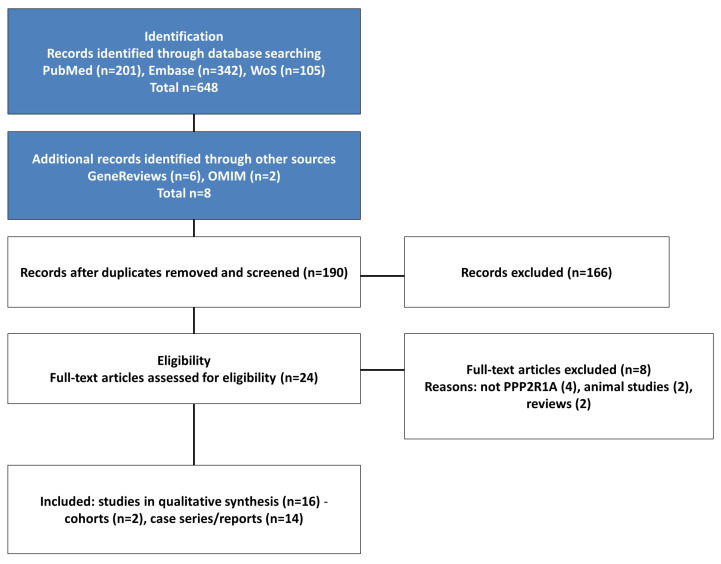
PRISMA 2020 flow diagram. Database records identified: PubMed (*n* = 201), Embase (*n* = 342), Web of Science (*n* = 105) (total *n* = 648). Additional sources: GeneReviews (*n* = 6), OMIM (*n* = 2) (total *n* = 8). After deduplication, records screened (*n* = 190); records excluded (*n* = 166). Full-text articles assessed (*n* = 24); full-text articles excluded (*n* = 8): not PPP2R1A (*n* = 4), animal studies (*n* = 2), reviews (*n* = 2). Studies included in qualitative synthesis (*n* = 16): cohorts (*n* = 2), case series/reports (*n* = 14).

The systematic search yielded 656 total records (648 from databases, 8 from additional sources). After removing 466 duplicates, 190 unique records were screened by title and abstract, of which 166 were excluded. Twenty-four full-text articles were assessed for eligibility, with 8 excluded for not meeting inclusion criteria (reasons: not *PPP2R1A* (n = 4), animal studies (n = 2), reviews (n = 2)). Ultimately, 16 studies were included in the qualitative synthesis (Figure 1).

### 2.5. Data Extraction and Quality Assessment

Data extraction was performed independently by two reviewers (J.Y. and A.A.) using a standardized, pre-piloted extraction form. Extracted variables included study characteristics (first author, year, country, study design, number of patients), genetic data (PPP2R1A variants reported in HGVS nomenclature, protein changes, zygosity, inheritance pattern, variant classification according to ACMG/AMP guidelines), clinical phenotype (developmental delay, intellectual disability, epilepsy, structural brain abnormalities, and other systemic manifestations), diagnostic methods, and patient outcomes when available.

For cohort studies, data were extracted at the study level with aggregate patient characteristics. For case reports and case series, data were extracted at the individual patient level to enable detailed phenotype analysis. Discrepancies in data extraction were resolved through discussion or consultation with a third reviewer (S.L.).

The methodological quality of included studies was assessed independently by two reviewers using the Joanna Briggs Institute (JBI) critical appraisal checklist for case reports, consisting of 8 standardized items. This checklist was applied uniformly to all included studies, as the predominantly case-based nature of *PPP2R1A* literature made this the most appropriate and consistent quality assessment tool across different study designs. Quality scores were used to contextualize findings during synthesis rather than for study exclusion. A comprehensive list of all extracted data points for each patient is available in Supplementary Material S2.

### 2.6. Data Synthesis

Due to the rarity of *PPP2R1A*-related disorders and the heterogeneity of study designs and outcome measures, quantitative meta-analysis was not feasible. Therefore, we conducted a structured narrative synthesis following established guidelines for systematic reviews without meta-analysis.

Data synthesis involved several components: (1) descriptive analysis of study characteristics (Table 1), (2) comprehensive mapping of the *PPP2R1A* mutational spectrum with variant classifications and functional predictions (Table 2), and (3) systematic tabulation of clinical feature frequencies across all reported patients (Table 3). For clinical feature analysis, denominators represent the number of patients for whom specific clinical information was available, as individual case reports varied in the completeness of phenotypic documentation.

Where possible, we explored patterns and relationships between genetic variants and clinical phenotypes, with particular attention to variant location within functional protein domains and corresponding clinical severity.

## 3. Results

### 3.1. Study Selection

The systematic search identified 656 records: 648 from database searches (PubMed *n* = 201, Embase *n* = 342, Web of Science *n* = 105) and 8 from additional sources (GeneReviews *n* = 6, OMIM *n* = 2). After removal of duplicates, 190 unique records were screened by title and abstract, of which 166 were excluded. Twenty-four full-text articles were assessed for eligibility, and eight were excluded for not meeting inclusion criteria (reasons: not PPP2R1A (n = 4), animal studies (n = 2), reviews (n = 2)). Ultimately, 16 studies met the inclusion criteria and were included in the qualitative synthesis, comprising 2 cohort studies and 14 case series/reports, representing a total of 60 patients with *PPP2R1A*-related neurodevelopmental disorders (Figure 1). The characteristics of included studies are summarized in Table 1.

### 3.2. Study Characteristics

The characteristics of the 16 included studies are summarized in Table 1. Publications spanned a decade from 2015 to 2025, with geographic representation across multiple continents including Europe, North America, Asia, and Australia. Study designs comprised predominantly case reports and case series (*n* = 14), with two cohort studies providing larger patient datasets.

Sample sizes and study types: Individual case reports predominated, while the largest contribution came from Lenaerts et al., who reported a multi-center cohort of 30 patients [1]. Baker et al. contributed a notable case series of 4 patients, several of whom presented with congenital heart defects, expanding the recognized systemic phenotype [5].

Diagnostic approaches: Whole-exome sequencing (WES) was the most frequently employed diagnostic method, utilized in both trio-based and proband-only configurations. Recent studies have increasingly incorporated whole-genome sequencing and prenatal sequencing approaches. Several studies employed complementary validation strategies: Qian et al. combined clinical exome sequencing with Sanger confirmation and functional validation for novel variants, demonstrating the importance of integrating genomic and functional evidence for variant classification [13].

Quality assessment: Using JBI critical appraisal criteria, 15 studies achieved high methodological quality scores, with 1 study rated as moderate quality, reflecting generally robust reporting standards across the literature.

### 3.3. Genetic Spectrum

Recurrent variants dominated the mutational spectrum. The most frequent variant was p.Arg182Trp (*n* = 12 patients), representing nearly 20% of all cases. Other common recurrent variants included p.Met180Thr (*n* = 6 patients) and p.Arg183Gln (*n* = 5 patients). All variants were detected through next-generation sequencing approaches, including whole-exome sequencing (14 studies), targeted gene panel sequencing (1 study), and whole genome sequencing (1 study), with Sanger sequencing confirmation. Variant pathogenicity was uniformly assessed according to ACMG/AMP guidelines using in silico prediction tools including CADD, PolyPhen-2, SIFT, and MutationTaster.

**Figure 2:**
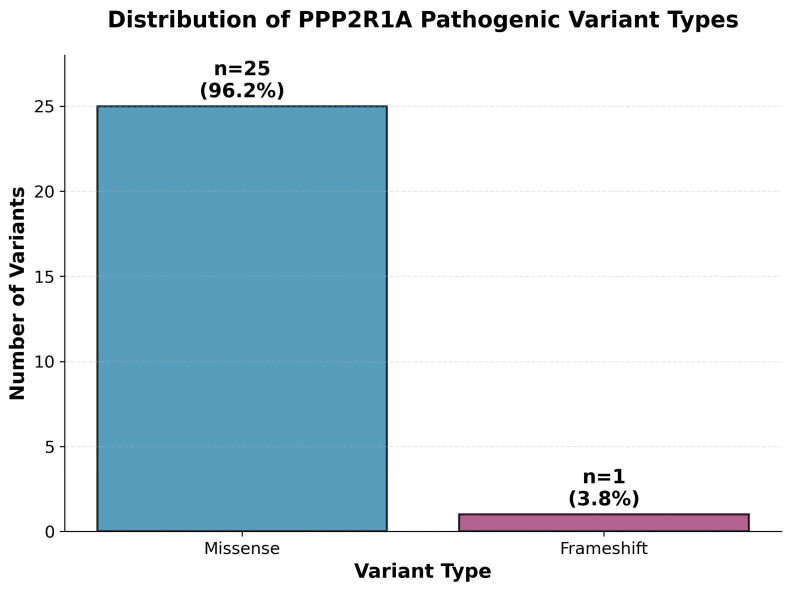
Distribution of *PPP2R1A* pathogenic variant types. Bar chart showing the predominance of missense variants (*n* = 25, 96.2%) over frameshift variants (*n* = 1, 3.8%) among the 26 distinct pathogenic variants identified in this systematic review. The overwhelming majority of variants are missense changes affecting critical functional domains, consistent with dominant-negative or gain-of-function mechanisms.

The mutational spectrum was overwhelmingly dominated by missense variants (*n* = 25, 96.2%), with only one frameshift variant (p. Asp282Argfs*14, 3.8%) identified (Figure 2). All missense variants were heterozygous and predominantly de novo, affecting critical amino acid residues within the protein structure. This predominance of missense changes supports a dominant-negative or gain-of-function pathogenic mechanism rather than haploinsufficiency.

**Figure 3:**
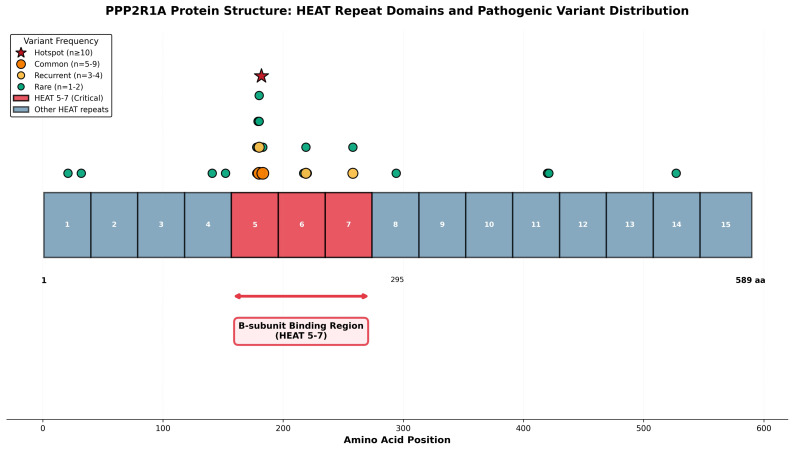
PPP2R1A protein structure and pathogenic variant distribution. Linear schematic representation of the PPP2R1A scaffolding A subunit (589 amino acids) showing 15 HEAT repeat domains (labeled 1–15). HEAT repeats 5–7 (shown in red) represent the critical B-subunit binding region, where 77% of all pathogenic variants cluster. All 26 pathogenic variants identified in this systematic review are mapped to their respective positions along the protein. Variant symbols are sized and colored by recurrence frequency: red stars indicate hotspots (n ≥ 10), orange circles indicate common variants (*n* = 5–9), yellow circles indicate recurrent variants (*n* = 3–4), and green circles indicate rare variants (*n* = 1–2). Notable hotspots include p.Arg182Trp (*n* = 12) at position 182 and p.Arg183Gln (*n* = 5) at position 183, both within HEAT repeat 5. The spatial clustering of variants within HEAT 5–7 highlights the critical functional importance of the B-subunit binding interface in disease pathogenesis. The B-subunit binding region is annotated below the protein structure with a bidirectional arrow spanning amino acids 157–273.

Spatial analysis revealed striking clustering of pathogenic variants within HEAT repeats 5–7 (Figure 3), with 20 of 26 variants (77%) localized to this critical B-subunit binding interface. The most frequent variant, p.Arg182Trp (*n* = 12), and the lethal p.Arg183Gln variant (*n* = 5) both reside within HEAT repeat 5 at positions 182–183, establishing this region as a mutational hotspot. Additional recurrent variants at position 180 (p.Met180Thr, *n* = 6; p.Met180Val, *n* = 4) and position 258 (p.Arg258His, *n* = 4) further underscore the functional importance of specific residues within the B-subunit binding domain. Clear genotype–phenotype relationships emerged, with HEAT5 variants (p.Arg182Trp, p.Arg183Gln) associated with severe phenotypes and increased mortality, while p.Arg258His (*n* = 4 patients, HEAT7) demonstrated comparatively milder courses, suggesting that variant position within different HEAT repeats influences functional impact.

Table 4 highlights the distinct severity gradient across *PPP2R1A* variants, presenting detailed clinical characteristics for all recurrent variants occurring in two or more individuals (n ≥ 2). The nine recurrent variants in Table 4 represent 44 of 60 patients (73% of our cohort), providing statistically meaningful correlation data demonstrating clear domain-specific patterns that guide clinical prognostication. Severity classifications are based on overall clinical presentation: Lethal phenotypes show mortality greater than 50 percent with profound impairment and life-threatening complications; severe phenotypes demonstrate significant multi-system involvement with profound developmental delay or intellectual disability; moderate phenotypes show moderate developmental impairment with variable systemic features; and milder phenotypes present with mild to moderate developmental delay and fewer systemic complications. Epilepsy prevalence and other percentages are calculated from reported cases with available data for each variant. Brain malformations include corpus callosum abnormalities, ventriculomegaly, cerebellar hypoplasia, and cortical malformations. Mortality rates reflect reported deaths during variable follow-up periods across studies.

The table demonstrates clear domain-specific severity patterns. HEAT5 variants (p.Arg182Trp, p.Arg183Gln, p.Met180Thr, p.Met180Val, p.Met180Arg, p.Pro179Leu) consistently show severe to lethal phenotypes with high epilepsy rates ranging from 67 to 100 percent and significant mortality risk. In contrast, HEAT7 variants (p.Arg258His) demonstrate relatively milder clinical courses with lower epilepsy rates (25 percent), better developmental outcomes (mild to moderate developmental delay rather than profound intellectual disability), and lower mortality. This pattern strongly supports the critical role of the B-subunit binding interface in disease pathogenesis, with disruption of the core HEAT5 binding surface producing more severe PP2A dysfunction than peripheral HEAT7 alterations.

Single occurrence variants (*n* = 1) are not included in the correlation table due to there being insufficient data to establish reliable genotype–phenotype patterns from individual cases. However, all 26 distinct pathogenic variants are comprehensively detailed in Table 2, and individual patient-level phenotypic data for all 60 cases are available in the Supplementary Material S2 Data Extraction Table, ensuring complete transparency regarding the full mutational spectrum.

### 3.4. Clinical Phenotypes

The clinical phenotype analysis across 60 patients revealed a consistent spectrum of neurodevelopmental features, with frequencies summarized in Table 3.

Core neurodevelopmental features were nearly universal. Developmental delay and intellectual disability were documented in all patients with available data (58/58). The severity ranged from mild to profound, with many patients requiring lifelong supportive care [1].

Epilepsy was present in approximately 50.9% of patients (29/57), with notable characteristics including early onset (often neonatal or infantile), treatment resistance, and progression to epileptic encephalopathy in severe cases. The Ruxmohan case exemplified the therapeutic challenges, demonstrating medically refractory seizures despite multiple interventions including polytherapy, ketogenic diet, and cannabidiol [19]. Lee et al. similarly documented treatment-resistant epilepsy requiring multiple antiepileptic medications in their Korean patient [22].

Structural brain abnormalities were identified in 83.1% of patients (49/59), with corpus callosum abnormalities (agenesis or hypoplasia; ACC/CC) being the most frequent finding (40.7%, 24/59). These ranged from complete agenesis to hypoplasia. Ventriculomegaly was documented in 32.2% of cases (19/59), often severe enough to require neurosurgical intervention. Cerebellar and brainstem hypoplasia occurred in 15.3% of patients (9/59), with some cases presenting as pontocerebellar hypoplasia-like phenotypes, expanding the recognized neuroanatomical spectrum.

Systemic manifestations included hypotonia in 60.3% of patients (35/58), often severe and contributing to feeding difficulties and motor delays. Congenital heart defects were documented in 8.6% of cases (5/58), with Baker et al. reporting multiple cardiac anomalies including pulmonary vein stenosis, coarctation, and septal defects, particularly in patients with the lethal p.Arg183Gln variant [5].

Genotype–phenotype correlations emerged from the data analysis. Patients with p.Arg182Trp and p.Arg183Gln variants consistently presented with severe phenotypes and increased mortality risk, while those with p.Arg258His variants demonstrated comparatively milder courses with better developmental outcomes and lower seizure rates.

Mortality and prognosis varied significantly by variant type. The p.Arg183Gln variant was associated with particularly poor outcomes, with Baker et al. and Wallace et al. documenting neonatal deaths in multiple cases, often related to severe cardiac and neurological complications [5,14].

Recent studies have significantly expanded the prenatal phenotypic spectrum of *PPP2R1A*-related disorders. Core prenatal features include ventriculomegaly, corpus callosum agenesis or hypoplasia, and aqueductal stenosis patterns. Hu et al. synthesized 12 prenatally diagnosed cases and documented ventriculomegaly in 92% (11/12), corpus callosum abnormalities in 50% (6/12), and congenital cardiac anomalies in 42% (5/12), establishing these as high-yield sonographic indicators [15].

Diagnostic utility of prenatal exome sequencing has been demonstrated across multiple studies. Wei et al. reported a diagnostic yield of 46.2% among CMA-negative fetuses with these imaging findings, supporting the clinical value of targeted prenatal WES [16]. Lei et al. identified two fetuses with recurrent HEAT5–6 variants (p.Arg182Trp and p.Arg183Trp) through trio-WES, confirming that hotspot variants are detectable prenatally [18].

Quantitative prenatal markers have been established by Hamill et al., who documented specific measurements including corpus callosum lengths of 20–26 mm (well below gestational norms) and third-ventricle diameters of 3–6 mm, consistent with aqueductal-type ventriculomegaly [17]. These objective parameters provide standardized criteria for targeted genetic testing.

Postnatal correlates support the prenatal findings. Wallace et al.’s detailed neonatal imaging (severe ventriculomegaly, absent callosal fibers, pontine hypoplasia) mirrors prenatal observations [14], while Lee et al. described a Dandy–Walker continuum pattern, expanding the recognized neuroimaging spectrum [22]. These postnatal cases validate the sonographic markers identified in fetal studies.

Genotype–phenotype correlations revealed significant variability, particularly for the p.Arg258His variant. While most patients with p.Arg258His demonstrated comparatively milder clinical courses with longer survival and less severe developmental impairment than those with HEAT5 hotspot variants, notable exceptions exist that challenge this generalization.

The case reported by Melas et al. exemplifies this phenotypic variability, describing a patient with p.Arg258His who presented with severe systemic manifestations including progressive microcephaly, profound growth deficiency, craniosynostosis, progressive scoliosis, and severe developmental delay [3]. This case expanded the recognized phenotypic spectrum associated with p.Arg258His, demonstrating that even variants typically associated with milder phenotypes can occasionally result in multisystem disease.

These findings underscore the importance of individualized prognostic counseling and highlight that variant position alone may not fully predict clinical severity. Additional genetic, epigenetic, or environmental modifiers may contribute to the observed phenotypic heterogeneity within the same variant group.

## 4. Discussion

### 4.1. Summary of Findings

This systematic review represents the most comprehensive synthesis of *PPP2R1A*-related neurodevelopmental disorders to date, encompassing 16 studies and 60 patients across four continents. While previous reviews, including the narrative review by Verbinnen et al. (2021) [20] and the landmark cohort by Lenaerts et al. (2021) [1], provided valuable insights, our PRISMA-compliant systematic approach advances the field by capturing 26 distinct variants—including novel variants reported after 2021 and prenatal cases—and challenges the earlier perception of uniformly poor outcomes by documenting milder phenotypes associated with specific variants such as p.Arg258His.

Our analysis revealed universal developmental delay and intellectual disability (100%, 58/58), but significant phenotypic variability in other features. Epilepsy emerged as a major comorbidity in approximately half of patients (50.9%, 29/57), often presenting with treatment-resistant seizures that significantly impact clinical management. Structural brain abnormalities were present in 83.1% (49/59) of cases, with corpus callosum abnormalities (40.7%) and ventriculomegaly (32.2%) being the most frequent findings. Importantly, head circumference showed bimodal distribution, with both microcephaly (17.2%) and macrocephaly (25.9%) reported, highlighting the heterogeneous nature of the disorder.

Phenotypic characterization revealed a broader clinical spectrum than previously recognized. While developmental delay was universal (100%), we documented significant variability in other features: Epilepsy emerged as a major comorbidity, often presenting with treatment-resistant seizures that significantly impact clinical management. Similarly, the high prevalence of structural brain abnormalities underscores the importance of early neuroimaging in diagnosis. Importantly, our analysis challenged the historical perception of uniformly poor outcomes, identifying milder phenotypes associated with specific variants, particularly p.Arg258His [1,3,5].

Diagnostic advances were particularly notable in prenatal medicine. Recent studies established objective imaging criteria, highlighting ventriculomegaly and corpus callosum abnormalities as hallmark features in prenatal detection. These findings have transformed *PPP2R1A*-related disorders from a primarily postnatal diagnosis to a recognizable prenatal condition, with direct implications for reproductive counseling and family planning [15,16,17,18].

Geographic representation across Europe, North America, Asia, and Australia suggests these disorders occur across diverse populations, though ascertainment bias toward more severe phenotypes in published literature remains a consideration. The high diagnostic yield of whole-exome sequencing in suspected cases (>90% when characteristic features are present) supports its clinical utility [4,13,16].

### 4.2. Genotype–Phenotype Correlations

Our systematic analysis established clear mechanistic relationships between variant location and clinical severity, providing insights into PP2A holoenzyme function and dysfunction. The clear correlation we identified provides direct clinical guidance; for instance, the presence of HEAT5 variants like p.Arg182Trp or p.Arg183Gln should alert clinicians to a high risk of severe outcomes, including early mortality and treatment-resistant epilepsy, prompting intensive multidisciplinary care planning from diagnosis. Biochemical studies demonstrate that these variants severely impair B-subunit binding, particularly affecting B55α interactions that are crucial for neuronal PP2A function [1,5,8,14].

Molecular mechanisms underlying these correlations involve disruption of specific protein–protein interactions within the PP2A holoenzyme complex. Variants affecting residues 180–183 (HEAT5) interfere with regulatory B-subunit binding, while position 258 (HEAT7) variants may retain partial function through alternative interaction mechanisms. Functional validation studies using super-resolution microscopy have revealed that pathogenic variants cause abnormal protein aggregation and reduced cellular expression, providing direct evidence of impaired protein homeostasis [1,2,13].

Residue-specific patterns emerged from our analysis, with Met180 substitutions showing unique associations with macrocephaly and reduced seizure frequency compared to adjacent Arg182/183 variants. This suggests that even within the same functional domain, individual amino acid positions contribute differently to holoenzyme stability and clinical phenotype [13].

Variable expressivity was most pronounced for p.Arg258His variants, where the majority showed milder developmental trajectories, yet severe exceptions exist. This phenotypic heterogeneity likely reflects the complex interplay between variant-specific biochemical effects and additional genetic or environmental modifiers, highlighting the limitations of genotype-based prognosis in individual cases [1,3].

Therapeutic implications of these correlations suggest that understanding the specific molecular defect may guide targeted interventions. Variants with preserved partial function (such as some HEAT7 changes) might be more amenable to pharmacological enhancement of residual PP2A activity, while complete loss-of-function variants may require alternative therapeutic strategies.

### 4.3. Clinical Implications

Our findings provide evidence-based guidance for postnatal clinical management. All patients with pathogenic PPP2R1A variants should receive comprehensive cardiac evaluation, including echocardiography, particularly those harboring p.Arg183Gln variants which showed high rates of congenital heart defects in multiple studies [5]. The established genotype–phenotype correlations facilitate variant interpretation in diagnostic sequencing. The identification of a HEAT5 variant, such as p.Arg182Trp or p.Arg183Gln, should prompt immediate and comprehensive clinical action, including a baseline echocardiogram to screen for congenital heart defects, continuous EEG monitoring for early seizure detection, and proactive planning for aggressive, multidisciplinary supportive care. Conversely, the presence of a p.Arg258His variant may allow for a more reassuring prognosis during genetic counseling, though careful monitoring remains essential [1,8,14].

For prenatal medicine, our systematic analysis establishes clear imaging criteria for targeted genetic testing. Fetuses presenting with ventriculomegaly, corpus callosum abnormalities, or aqueductal stenosis should be considered for trio-based whole-exome sequencing after normal karyotype and chromosomal microarray [15,18]. The high diagnostic yield (92% ventriculomegaly, 50% corpus callosum abnormalities in confirmed cases) supports the clinical utility of this approach. When cardiac anomalies co-occur with brain malformations, *PPP2R1A* should be prioritized in the differential diagnosis [16].

Genetic counseling must address the broad phenotypic spectrum and variant-specific prognosis. The documented cases of pregnancy termination following prenatal PPP2R1A diagnosis highlight the significant impact of molecular diagnosis on reproductive decision-making [17,18]. Counselors should emphasize that while severe variants like p.Arg183Gln carry high mortality risk, the spectrum includes milder phenotypes, particularly with p.Arg258His variants [1,3]. This variability necessitates individualized counseling based on specific variant characteristics and functional evidence.

Long-term clinical management should anticipate variant-specific complications. Patients with HEAT5 variants require aggressive seizure management, as treatment-resistant epilepsy is common [19,22]. Regular developmental assessments and early intervention services are essential across the spectrum. The recognition of *PPP2R1A*-related disorders in prenatal and neonatal periods enables proactive multidisciplinary care planning and family support [1].

### 4.4. Therapeutic Implications and Clinical Management

While no *PPP2R1A*-specific targeted therapies currently exist, our systematic review provides important evidence-based guidance for clinical management and reveals potential therapeutic avenues.

Seizure management: The high prevalence of epilepsy (50.9%) and its association with severe outcomes, particularly in HEAT5 variants (p.Arg182Trp, p.Arg183Gln, p.Met180Thr), necessitates early and aggressive antiepileptic therapy. Many patients required multiple antiepileptic drugs for seizure control, and some cases remained refractory despite polytherapy. Variant-specific prognostic information may guide treatment intensity and counseling regarding realistic expectations for seizure control.

Developmental interventions: Given universal developmental delay and intellectual disability (100%), early initiation of comprehensive developmental intervention programs is essential. These should include physical therapy, occupational therapy, and speech–language therapy tailored to individuals’ needs. The variant-specific severity differences we identified (severe HEAT5 vs. relatively milder HEAT7 variants) can inform realistic goal setting and resource allocation for intervention programs.

Genetic counseling and reproductive planning: The predominance of de novo variants (>95% of cases) provides reassuring recurrence risk counseling for families, with empiric recurrence risk estimated at <1% in the absence of parental mosaicism. However, parental testing for low-level mosaicism should be considered, particularly for parents planning additional pregnancies. Prenatal diagnosis is technically feasible for known familial variants.

Surveillance protocols: Regular multidisciplinary surveillance is warranted given the high frequency of multi-system involvement. Recommended monitoring includes: (1) neuroimaging (MRI) to assess for progressive brain malformations or acquired changes; (2) growth and nutritional assessment given frequent feeding difficulties (>40% of cases); (3) serial EEG monitoring for epilepsy development or progression; and (4) developmental assessments to guide intervention strategies. Variant-specific surveillance intensity may be appropriate, with more intensive monitoring for HEAT5 variants associated with higher mortality risk.

Future therapeutic strategies: The mechanistic insight that pathogenic variants predominantly disrupt the critical B-subunit binding interface (HEAT repeats 5–7) suggests potential therapeutic approaches. PP2A activator compounds or allosteric modulators that stabilize the disrupted holoenzyme complex represent rational therapeutic targets, though these remain experimental. Additionally, understanding the dominant-negative mechanism suggests that approaches to reduce mutant protein expression (e.g., antisense oligonucleotides or RNA interference) might restore normal PP2A function. However, such strategies require extensive preclinical validation before clinical application.

Precision medicine approach: The clear genotype–phenotype correlations we identified support a precision medicine approach to *PPP2R1A*-related disorders. Variant-specific prognosis can guide clinical decision-making, family counseling, and end-of-life care discussions, particularly for severe HEAT5 variants with high mortality rates. As additional cases are reported and long-term outcome data accumulate, variant-specific clinical guidelines may become feasible.

### 4.5. Limitations

This review has limitations. First, the included studies were predominantly case reports and small series, introducing publication bias and limiting generalizability. Second, phenotypic data were variably reported, with missing information on cognitive, behavioral, or systemic features in several cases. Third, functional studies remain limited, restricting our ability to fully explain genotype–phenotype correlations. Finally, due to heterogeneity and small sample sizes, meta-analysis was not feasible, and findings rely on narrative synthesis.

### 4.6. Future Directions and Concluding Remarks

Future research must move beyond descriptive studies. Establishing a global, multicenter patient registry is a critical next step to conduct prospective natural history studies, which are essential for defining clinical endpoints for future therapeutic trials. Furthermore, given that the core pathomechanism involves disruption of the PP2A holoenzyme, translational research should focus on exploring the potential of small-molecule PP2A modulators. Importantly, future therapeutic approaches should explicitly adopt a variant-stratified clinical trial design. Patients with hotspot variants (e.g., HEAT5 vs. HEAT7) may require differential enrollment or monitoring, thereby increasing the likelihood of detecting meaningful therapeutic effects. Such precision trial frameworks are essential to advance *PPP2R1A*-targeted therapy. Roldán et al. demonstrated the value of cellular approaches, providing direct evidence that atypical *PPP2R1A* variants impair protein homeostasis through reduced immunofluorescence intensity and abnormal cytoplasmic protein aggregates visualized by super-resolution microscopy [2]. Integration of prenatal, pediatric, and adult cases will be essential to capture the full phenotypic spectrum and inform long-term prognostic counseling.

Taken together, our findings indicate that *PPP2R1A*-related neurodevelopmental disorders encompass a broad and heterogeneous spectrum, ranging from lethal neonatal disease to survivable forms with variable cognitive and neurological outcomes. Recognition of recurrent hotspot variants and their associated phenotypes can facilitate diagnosis, prognostication, and counseling. While most affected patients exhibit profound developmental impairment, milder courses are possible, highlighting the need for careful genotype–phenotype correlation. Continued collaboration across centers will be crucial to refine our understanding and ultimately explore potential therapeutic avenues.

## Figures and Tables

**Table 1:** Characteristics of included studies reporting *PPP2R1A*-related neurodevelopmental disorders.

First Author (Year)	Country	Study Design	Study Type	Number of Patients	Quality
**Baker EK (2022) [5]**	USA/Israel	Case series	Case series	4	High
**Fitzgerald, TW (2015) [4]**	UK and Republic of Ireland	Cohort Study	Large-scale genomic analysis study	3	High
**Hamill, C (2025) [17]**	Australia	Case series	Case Series	3	Moderate
**Houge G (2015) [6]**	UK, Netherlands, Israel, Norway	Case Series	Case Series	5	High
**Hu, J (2025) [15]**	China	Case report	Case report	1	High
**Lee, J (2023) [22]**	Korea	Case report	Case report	1	High
**Lei T. (2023) [18]**	China	Case Report	Case Report	1	High
**Lenaerts L (2021) [1]**	Multi-country	Cohort study	Case series	30	High
**Melas, M (2021) [3]**	USA	Case Report	Case Report	1	High
**Qian Y (2023) [13]**	China	Case Series	Case Series	5	High
**Roldán, M (2023) [2]**	Spain	Case Report	Case Report	2	High
**Ruxmohan, S (2021) [19]**	USA	Case Report	Case Report	1	High
**Wallace, A (2019) [14]**	United States	Case Report	Case Report	1	High
**Wei, X (2024) [16]**	China	Case Series	Case Report	1	High
**Yanghui Zhang (2020) [23]**	China	Case Report	Case Report	1	High
**Zarante-Bahamon, (2025) [24]**	Colombia	Case Report	Case Report	1	High

This table summarizes the key features of the 16 included studies, including first author (year), country, study design and type (cohort, case series, case report), number of patients (N) contributed by each study, and study quality.

**Table 2:** *PPP2R1A* variants reported in the included studies.

cDNA Change (HGVS)	Protein Change (HGVS)	HEAT Repeat Domain	*n*	Notes
**c.61C>T**	p.Arg21Cys	HEAT 1	1	Novel variant, reported in Roldán 2023 [2]
**c.96C>G**	p.Ile32Met	HEAT 1	1	Lenaerts 2021 cohort [1]
**c.421T>A**	p.Phe141Ile	HEAT 4	1	Lenaerts 2021 cohort [1]
**c.455C>T**	p.Ser152Phe	HEAT 4	1	Lenaerts 2021 cohort [1]
**c.532A>T**	p.Thr178Ser	HEAT 5	1	Lenaerts 2021 cohort [1]
**c.533C>A**	p.Thr178Asn	HEAT 5	1	Lenaerts 2021 cohort [1]
**c.536C>A**	p.Pro179His	HEAT 5	1	Baker EK 2022 [5]
**c.536C>T**	p.Pro179Leu	HEAT 5	3	Lenaerts 2021 cohort [1]
**c.538A>G**	p.Met180Val	HEAT 5	4	Includes Qian 2023 [13]
**c.539T>C**	p.Met180Thr	HEAT 5	6	Includes Qian 2023, Lenaerts 2021 [1,13]
**c.539T>A**	p.Met180Lys	HEAT 5	1	Lenaerts 2021 cohort [1]
**c.539T>G**	p.Met180Arg	HEAT 5	2	Includes Roldán 2023 [2]
**c.544C>T**	p.Arg182Trp	HEAT 5	12	Most frequent variant, recurrent hotspot
**c.547C>T**	p.Arg183Trp	HEAT 5	1	Lenaerts 2021 cohort [1]
**c.548G>A**	p.Arg183Gln	HEAT 5	5	Lethal variant, cardiac phenotype (Baker EK 2022) [5]
**c.650A>G**	p.Gln217Arg	HEAT 6	1	Lee J 2023 [22]
**c.655T>C**	p.Ser219Pro	HEAT 6	1	Zarante-Bahamon A 2025 [24]
**c.656C>T**	p.Ser219Leu	HEAT 6	4	Includes Lenaerts 2021 [1]
**c.658G>A**	p.Val220Met	HEAT 6	4	Lenaerts 2021 cohort [1]
**c.772C>A**	p.Arg258Ser	HEAT 7	1	Lenaerts 2021 cohort [1]
**c.773G>A**	p.Arg258His	HEAT 7	4	Includes Houge 2015 [6]
**c.843dupA**	p.Asp282Argfs*14	HEAT 7	1	Qian 2023 [13]
**c.1409T>C**	p.Val470Ala	HEAT 12	1	Qian 2023 [13]
**c.1493G>T**	p.Arg498Leu	HEAT 13	1	Qian 2023 [13]
**c.1565C>T**	p.Pro522Leu	HEAT 14	1	Lei T 2023 [18]

(cDNA and protein changes in HGVS nomenclature; *N* = number of reported patients). Abbreviations: HGVS = Human Genome Variation Society; *N* = number of patients; HEAT = Huntingtin, Elongation factor 3 (EF3), protein phosphatase 2A (PP2A) A subunit, TOR1

**Table 3:** Clinical features of patients with *PPP2R1A*-related neurodevelopmental disorders (n/N, %).

Clinical Feature	n/N (%)	Notes
**Global developmental delay/Intellectual disability**	58/58 (100%)	Reported in all patients
**Seizures/Epilepsy**	29/57 (50.9%)	Frequently onset in neonatal/infantile period
**Structural brain abnormalities (overall)**	49/59 (83.1%)	ACC/CC hypoplasia, ventriculomegaly, delayed myelination
**Agenesis/hypoplasia of corpus callosum (ACC/CC)**	24/59 (40.7%)	Most common structural abnormality
**Ventriculomegaly/Hydrocephalus**	19/59 (32.2%)	Some cases associated with lethal outcomes
**Cerebellar/brainstem hypoplasia**	9/59 (15.3%)	Often observed in severe phenotypes
**Hypotonia**	35/58 (60.3%)	Present from neonatal period
**Microcephaly**	10/58 (17.2%)	Both microcephaly and macrocephaly reported
**Macrocephaly**	15/58 (25.9%)	Both microcephaly and macrocephaly reported
**Dysmorphic features**	31/58 (53.4%)	Including facial dysmorphism, strabismus, high palate
**Congenital heart defects (CHD)**	5/58 (8.6%)	Highlighted in Baker EK study; associated with lethal p.Arg183Gln
**Other systemic features**	Variable	Visual impairment, hearing loss, skeletal anomalies, renal/genital malformations

Abbreviations: *n* = number of affected patients; *N* = number of patients with available data for that feature; ACC/CC = agenesis/hypoplasia of the corpus callosum; CHD = congenital heart defects.

**Table 4:** Genotype–phenotype Correlations for Recurrent *PPP2R1A* Variants (*n* ≥ 2).

Variant	cDNA	Domain	*n*	Severity	Epilepsy	Developmental Outcome	Brain Malformations	Mortality	Key Features
p.Arg182Trp	c.544C>T	HEAT5	12	Severe	70%	Profound DD/ID	Frequent	Moderate	Most frequent variant, early-onset seizures, microcephaly
p.Met180Thr	c.539T>C	HEAT5	6	Severe	67%	Moderate-severe DD/ID	Common	Moderate	Feeding difficulties, growth impairment
p.Arg183Gln	c.548G>A	HEAT5	5	Lethal	100%	Severe DD/ID	Universal	80%	Cardiac involvement, refractory seizures, highest mortality
p.Met180Val	c.538A>G	HEAT5	4	Severe	75%	Moderate–severe DD/ID	Common	Low–moderate	Variable severity within cohort
p.Arg258His	c.773G>A	HEAT7	4	Milder	25%	Mild–moderate DD/ID	Less common	Low	Relatively favorable prognosis, better developmental outcomes
p.Ser219Leu	c.656C>T	HEAT6	4	Severe	75%	Severe DD/ID	Present	Not reported	CCA, hypotonia; epilepsy common
p.Val220Met	c.658G>A	HEAT6	4	Moderate	50%	Moderate DD/ID	Universal	Not reported	CCA, hypotonia
p.Pro179Leu	c.536C>T	HEAT5	3	Severe	67%	Severe DD/ID	Common	Moderate	Microcephaly, corpus callosum abnormalities
p.Met180Arg	c.539T>G	HEAT5	2	Severe	100%	Severe DD/ID	Present	Not reported	Very limited data, severe presentation

Note: DD = developmental delay; ID = intellectual disability; CCA = corpus callosum abnormalities. Percentages calculated from available data for each variant. Severity classifications based on overall clinical presentation, developmental outcomes, and multi-system involvement. Mortality rates reflect reported deaths during follow-up periods (variable duration across studies).

## Data Availability

No new data were created in this study. All data supporting the reported results were extracted from previously published articles, which are cited in the References section. Extracted datasets and summary tables generated during this study are available from the corresponding author upon reasonable request.

## Supplementary Materials

The following supporting information can be downloaded at: https://www.mdpi.com/article/10.3390/genes16121508/s1, Supplementary Material S1: Supplementary Methods; Supplementary Material S2: Data_Extraction_Table.

## Abbreviations

The following abbreviations are used in this manuscript:

ACC/CC	agenesis/hypoplasia of the corpus callosum
ACMG/AMP	American College of Medical Genetics and Genomics/Association for Molecular Pathology
CHD	Congenital heart defects
CMA	Chromosomal microarray analysis
DD	Developmental delay
HEAT	Huntingtin, Elongation factor 3 (EF3), protein phosphatase 2A (PP2A) A subunit, TOR1
HGVS	Human Genome Variation Society
HJS2	Houge-Janssens syndrome 2
ID	Intellectual disability
JBI	Joanna Briggs Institute
NDD	Neurodevelopmental disorder
OMIM	Online Mendelian Inheritance in Man
PP2A	Protein phosphatase 2A
PRISMA	Preferred Reporting Items for Systematic Reviews and Meta-Analyses
VUS	Variant of uncertain significance
WES	Whole-exome sequencing
WGS	Whole genome sequencing

## References

[1] Lenaerts L., Reynhout S., Verbinnen I., Laumonnier F., Toutain A., Bonnet-Brilhault F., Hoorne Y., Joss S., Chassevent A.K., Smith-Hicks C. (2021). The broad phenotypic spectrum of PPP2R1A-related neurodevelopmental disorders correlates with the degree of biochemical dysfunction. Genet. Med..

[2] Roldán M., Nolasco G.A., Armengol L., Frías M., Morell M., García-Aragonés M., Epifani F., Muchart J., Ramírez-Almaraz M.L., Martorell L. (2023). Advanced Optical Microscopy: Unveiling Functional Insights Regarding a Novel PPP2R1A Variant and Its Unreported Phenotype. Int. J. Mol. Sci..

[3] Melas M., Mathew M.T., Mori M., Jayaraman V., Wilson S.A., Martin C., Jacobson-Kelly A.E., Kelly B.J., Magrini V., Mardis E.R. (2021). Somatic variation as an incidental finding in the pediatric next-generation sequencing era. Cold Spring Harb. Mol. Case Stud..

[4] (2015). The Deciphering Developmental Disorders Study. Large-scale discovery of novel genetic causes of developmental disorders. Nature.

[5] Baker E.K., Solivio B., Pode-Shakked B., Cross L.A., Sullivan B., Raas-Rothschild A., Chorin O., Barel O., Bar-Yosef O., Husami A. (2022). PPP2R1A neurodevelopmental disorder is associated with congenital heart defects. Am. J. Med. Genet. Part A.

[6] Houge G., Haesen D., Vissers L.E.L.M., Mehta S., Parker M.J., Wright M., Vogt J., McKee S., Tolmie J.L., Cordeiro N. (2015). B56δ-related protein phosphatase 2A dysfunction identified in patients with intellectual disability. J. Clin. Investig..

[7] Verbinnen I., Douzgou Houge S., Hsieh T.C., Lesmann H., Kirchhoff A., Geneviève D., Brimble E., Lenaerts L., Haesen D., Levy R.J. (2025). Pathogenic de novo variants in *PPP2R5C* cause a neurodevelopmental disorder within the Houge-Janssens syndrome spectrum. Am. J. Hum. Genet..

[8] Houge G.D., Houge S.D., Hsieh T.C., Verbinnen I., Janssens V. (2025). Houge-Janssens syndrome. Eur. J. Hum. Genet..

[9] Shang L., Henderson L.B., Cho M.T., Petrey D.S., Fong C.T., Haude K.M., Shur N., Lundberg J., Hauser N., Carmichael J. (2016). De novo missense variants in PPP2R5D are associated with intellectual disability, macrocephaly, hypotonia, and autism. Neurogenetics.

[10] Musumeci A., Vinci M., Verbinnen I., Treccarichi S., Nigliato E., Chiavetta V., Greco D., Vitello G.A., Federico C., Janssens V. (2025). PPP2R5E: New gene potentially involved in specific learning disorders and myopathy. Gene.

[11] Muir A.M., Reich A., Zou F., Carere D.A., Harasink S.M., Tran L., McGivern B. (2025). A recurrent variant in *PPP2R5C* identified in individuals with macrocephaly, intellectual disability, and seizures. Hum. Genet. Genom. Adv..

[12] Sandal P., Jong C.J., Merrill R.A., Kollman G.J., Paden A.H., Bend E.G., Sullivan J., Spillmann R.C., Shashi V., Vulto-van Silfhout A.T. (2024). De novo missense variants in the PP2A regulatory subunit PPP2R2B in a neurodevelopmental syndrome: Potential links to mitochondrial dynamics and spinocerebellar ataxias. Hum. Mol. Genet..

[13] Qian Y., Jiang Y., Wang J., Li G., Wu B., Zhou Y., Xu X., Wang H. (2023). Novel Variants of PPP2R1A in Catalytic Subunit Binding Domain and Genotype–Phenotype Analysis in Neurodevelopmentally Delayed Patients. Genes.

[14] Wallace A., Caruso P., Karaa A. (2019). A Newborn with Severe Ventriculomegaly: Expanding the PPP2R1A Gene Mutation Phenotype. J. Pediatr. Genet..

[15] Hu J., Pang J., Zhou L., Kuang H., Yu W., Peng Y. (2025). Prenatal Characterization of Houge-Janssens Syndrome Type 2: A Case Report and Systematic Review of Fetal Phenotypes Associated With PPP2R1A Mutations. Mol. Genet. Genom. Med..

[16] Wei X., Cai L., Zhang L., Chen J., Zhang Y., Meng M., Yang Y., Zhou X., Zou G., Sun L. (2024). Prenatal Diagnosed Agenesis of the Corpus Callosum: Identifying the Underlying Genetic Etiologies. Prenat. Diagn..

[17] Hamill C., Goergen S., Fahey M., Roscioli T., Gorrie A., Curd H., Gelfand N. (2025). The Prenatal Neuro-Radiological Phenotype Associated with a Recurrent Pathogenic Variant in PPP2R1A. Prenat. Diagn..

[18] Lei T., Zhen L., Yang X., Pan M., Fu F., Han J., Li L., Li D., Liao C. (2023). Prenatal Diagnosis of PPP2R1A-Related Neurodevelopmental Disorders Using Whole Exome Sequencing: Clinical Report and Review of Literature. Genes.

[19] Ruxmohan S., Quinonez J., Yadav R.S., Shrestha S., Poudel S., Stein J.D. (2021). Refractory Epilepsy in a Toddler With PPP2R1A Gene Mutation and Congenital Hydrocephalus. Cureus.

[20] Verbinnen I., Vaneynde P., Reynhout S., Lenaerts L., Derua R., Houge G., Janssens V. (2021). Protein Phosphatase 2A (PP2A) mutations in brain function, development, and neurologic disease. Biochem. Soc. Trans..

[21] Page M.J., McKenzie J.E., Bossuyt P.M., Boutron I., Hoffmann T.C., Mulrow C.D., Shamseer L., Tetzlaff J.M., Akl E.A., Brennan S.E. (2021). The PRISMA 2020 statement: An updated guideline for reporting systematic reviews. Syst. Rev..

[22] Lee J., Yoo J., Lee S., Lee J.W., Park E.G. (2023). PPP2R1A-Related Neurodevelopmental Disorder: The First Korean Case with a Novel Variant of PPP2R1A and Literature Review. Ann. Clin. Lab. Sci..

[23] Zhang Y., Li H., Wang H., Jia Z., Xi H., Mao X. (2020). A De Novo Variant Identified in the PPP2R1A Gene in an Infant Induces Neurodevelopmental Abnormalities. Neurosci. Bull..

[24] Zarante-Bahamon A.M., Cortés-Rojas M.C., Ramon-Gómez J.L. (2025). A de novo missense variant Ser219Pro in PPP2R1A leads to macrocephaly in Houge-Janssens syndrome type 2. Clin. Dysmorphol..

