# Supplementary material for: Clinical and Molecular Spectrum of PPP2R1A-Related Neurodevelopmental Disorders: A Systematic Review
Source: Genes (Basel). 2025 Dec 16;16(12):1508. doi: 10.3390/genes16121508 (PMC12733044; doi:10.3390/genes16121508)
Supplement: Supplementary file 1 [file genes-16-01508-s001.zip › supplementary material S1 - supplementary methods.pdf]

Supplementary Methods – Search Strategy

PubMed (searched up to March 2025):

("PPP2R1A"[All Fields] OR "protein phosphatase 2 regulatory subunit A alpha"[All Fields]) AND ("neurodevelopmental disorders"[MeSH Terms] OR "intellectual disability"[MeSH Terms] OR "epilepsy"[MeSH Terms] OR "brain abnormalities"[MeSH Terms] OR "neurodevelopmental disorder"[All Fields] OR "intellectual disability"[All Fields] OR "epilepsy"[All Fields] OR "brain malformations"[All Fields])

Embase (searched up to March 2025):

('ppp2r1a'/exp OR 'ppp2r1a':ab,ti OR 'protein phosphatase 2 regulatory subunit a alpha':ab,ti) AND ('neurodevelopmental disorder'/exp OR 'intellectual disability'/exp OR 'epilepsy'/exp OR 'brain malformation'/exp OR 'neurodevelopmental disorder':ab,ti OR 'intellectual disability':ab,ti OR 'epilepsy':ab,ti OR 'brain malformation':ab,ti)

Web of Science (searched up to March 2025):

TS=("PPP2R1A" OR "protein phosphatase 2 regulatory subunit A alpha") AND TS=("neurodevelopmental disorder" OR "intellectual disability" OR "epilepsy" OR "brain malformation")

Additional sources:

- Reference lists of GeneReviews (PPP2R1A-related neurodevelopmental disorder / Houge–Janssens syndrome 2)
- Reference lists of OMIM #616362
